# Retraction Note: Sciadopitysin mitigates spermatological and testicular damage instigated by paraquat administration in male albino rats

**DOI:** 10.1038/s41598-024-80085-y

**Published:** 2024-11-25

**Authors:** Muhammad Umar Ijaz, Mohammad Qamer, Ali Hamza, Hussain Ahmed, Tayyaba Afsar, Mahmoud Abulmeaty, Arusha Ayub, Suhail Razak

**Affiliations:** 1 Department of Zoology, Wildlife and Fisheries, University of Agriculture, Faisalabad, 38040 Pakistan; 2 Department of Zoology, The University of Buner, Buner, Khyber Pakhtunkhwa Pakistan; 3 Department of Community Health Sciences, College of Applied Medical Sciences, King Saud University, 11433 Riyadh, Saudi Arabia; 4 Department of Medicine, School of Health Sciences, University of Georgia, Tbilisi, GA Georgia

Retraction of: *Scientific Reports* 10.1038/s41598-023-46898-z, published online 13 November 2023

The Editors have retracted this Article. After publication, concerns were raised regarding areas of high similarity in the image presented in Fig. 3D. The Authors have been unable to share the raw data from this study upon request.

The Editors therefore no longer have confidence in the presented data.

Suhail Razak disagrees with the retraction. Muhammad Umar Ijaz, Ali Hamza, Tayyaba Afsar, Mahmoud Abulmeaty, and Arusha Ayub have not responded to correspondence from the Editors about this retraction. The Editors were not able to obtain current contact details of Mohammad Qamer and Hussain Ahmed.

